# Targeting Metabolic Reprogramming in Bladder Cancer Immunotherapy: A Precision Medicine Approach

**DOI:** 10.3390/biomedicines13051145

**Published:** 2025-05-09

**Authors:** Fuyang Liu, Kai Li, Qingyi Zhu

**Affiliations:** Department of Urology, The Second Affiliated Hospital of Nanjing Medical University, Nanjing 210011, China

**Keywords:** bladder cancer, immunotherapy, metabolic reprogramming, tumor microenvironment

## Abstract

Bladder cancer, as a highly heterogeneous malignant tumor of the urinary system, is significantly affected by tumor metabolic reprogramming in its response to immunotherapy. This review systematically elaborates on the molecular mechanisms of abnormal glucose and lipid metabolism in the bladder cancer microenvironment and immune escape, and discusses precision treatment strategies based on metabolic regulation. In the future, it will be necessary to combine spatiotemporal omics and artificial intelligence technologies to construct a multi-target intervention system for the metabolic–immune interaction network, promoting a paradigm shift in precision treatment for bladder cancer.

## 1. Introduction

Bladder cancer (BCa) is a common malignant lesion in the urinary system. In Asia, it is the eighth most common cancer among men. In 2022, an estimated 613,791 cases were reported worldwide [1]. BCa mainly includes muscle-invasive bladder cancer (MIBC) and non-muscle-invasive bladder cancer (NMIBC). Among them, NMIBC can lead to up to 75% of bladder cancers [2]. BCa is a group of different cancers, including at least 40 different histological subtypes, causing approximately 165,000 deaths worldwide each year [3]. Most histological subtypes of BCa are urothelial carcinoma (UC), previously known as transitional cell carcinoma, while up to 10% are non-urothelial carcinomas. The term “urothelial subtype” refers to a category of BCa that originates from urothelial cells but exhibits other and unique histological characteristics beyond normal UC. These differentiations include UC with mixed histological features, such as squamous or glandular differentiation, or micropapillary UC. In contrast, “non-urothelial subtype” describes bladder accessory cells that come from a completely different cell lineage, not from urothelial cells; they appear through other differentiation pathways and are different in their histological appearance and cellular origin, each showing different clinical and biological characteristics [4]. Thus, BCa is a highly heterogeneous malignant lesion.

**Figure 1:**
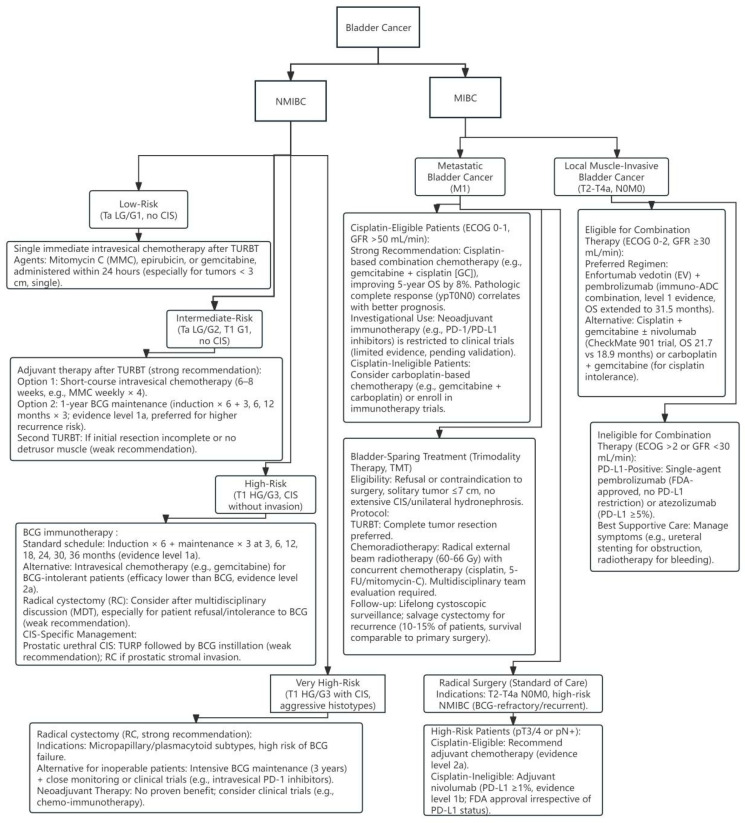
Bladder cancer treatment flowchart. Adapted from EAU Guidelines on Bladder Cancer [9,10].

For decades, the treatment prospects for BCa have remained relatively stagnant. Transurethral resection of bladder tumor (TURBT) combined with postoperative instillation therapy has become the standard treatment for NMIBC, while the main treatment for MIBC is cytotoxic chemotherapy [5]. Commonly used drugs include cisplatin and gemcitabine [6]. For advanced BCa, platinum-based drugs are not suitable for some patients (Figure 1). As interest in the molecular and histopathological heterogeneity of BCa gradually increases, there is an emphasis on the necessity of personalized treatment modalities for these tumors’ unique biological characteristics; various new drugs have begun to emerge, especially in the fields of immunotherapy and molecular targeted therapy. Immune checkpoint therapy (ICT) has become a new choice for patients with recurrence after platinum-based therapy or those who are not eligible for platinum-based therapy [6]. ICT targets cytotoxic T lymphocyte antigen 4 (CTLA4) and PD-1/PD-L1, activating the body’s immune response to kill tumor cells [7]. Although immune checkpoint inhibitors (ICI), such as programmed death (ligand) 1 (PD-1/PD-L1) monoclonal antibodies, have significantly improved the survival of some patients, the insufficient immune therapy response rate and drug resistance still limit its clinical effectiveness. Recent studies have found [8] that metabolic reprogramming (such as enhanced glycolysis, abnormal lipid metabolism) in the tumor microenvironment (TME) not only drives tumor progression, but also suppresses immune cell function through “metabolic competition”, shaping the core mechanism of the immunosuppressive microenvironment. Targeting metabolic pathways to reverse immune escape has become an important strategy in the era of precision medicine.

This article focuses on the interaction between metabolism and immunity in BCa, discussing how to improve the efficacy of immunotherapy through precise regulation of glucose and lipid metabolism.

## 2. Current Status of BCa Immunotherapy and Its Association with Metabolic Regulation

### 2.1. The Main Methods of Immunotherapy

#### 2.1.1. Cellular Immunotherapy

The human immune system has the capability to recognize and eliminate potentially harmful cells. However, most patients with advanced cancer are unable to mount an anti-tumor immune response. Cancer cells employ a variety of strategies to evade and weaken our defense mechanisms. Cellular immunotherapy is a treatment approach that involves modifying or activating the body’s own immune cells, enabling them to recognize and attack diseased cells. It involves various types of immune cells, including dendritic cells, natural killer cells, and T cells. Adoptive cell transfer therapy, such as tumor-infiltrating lymphocyte therapy (TILs), has developed multiple methods. Lymphocytes are extracted from tumor tissue, expanded in vitro, and then reinfused. TILs from primary bladder tumors can recognize tumor-associated antigens, as well as new antigens [11]. However, TILs require sufficiently large surgical samples and appropriate experimental facilities, and their future development remains to be observed. Through genetic engineering techniques, T cells are extracted from patients and equipped with “chimeric antigen receptors (CARs)”, thus developing CAR-T cell therapy, which has been applied to patients with blood cancers and has achieved a high overall response rate [12]. Nevertheless, CAR-T cell therapy has encountered some difficulties in exploring the treatment of bladder cancer, with clinical trials actively underway but not yet yielding the same successful outcomes [13]. Autologous and allogeneic natural killer cell adoptive cell transfer therapies are under investigation, showing potential efficacy for patients with solid tumors, but also presenting some issues [14]. Successfully expanding autologous NK cells from cancer patients to obtain a sufficient number of functional NK cells for reinfusion is challenging, and the response to tumor cells is also poor. Allogeneic NK cell therapy has shown better clinical outcomes than autologous NK cell therapy in treating breast cancer, but there are side effects [15]. Currently, NK cells have been designed to lyse 20 types of human cancer cell lines, including those of the genitourinary system, and the future prospects for research on bladder cancer treatment are promising [16].

Currently, one of the more mature forms of cellular immunotherapy is the intravesical instillation of bacillus Calmette-Guérin (BCG) therapy. Since its first introduction in 1976, this method has become a landmark in immunotherapy and is primarily used for the treatment of bladder malignancies. Compared to modern immune checkpoint inhibitors (ICIs), BCG therapy has been around much longer [17]. BCG is a strain of live-attenuated Mycobacterium bovis, primarily used as a vaccine against tuberculosis. Its antitumor activity against BCa consists of various mechanisms, and the immune system plays main role in response to both BCG and tumor antigens, and is capable of inducing T cell-dependent tumor-specific immunity [18]. Regarding the explanation of its antitumor mechanism, one theory posits that it acts as a substance interacting with urothelial cells, activating both innate and adaptive immune responses [19]. Today, BCG has become the cornerstone of medical treatment for patients. BCG bladder perfusion prevents the recurrence of NMIBC through local immune activation, although its efficacy against invasive cancer is limited.

#### 2.1.2. ICIs

Immunooncology is an ever-evolving field of drug development that involves the use of drugs to leverage the patient’s own immune system against cancer by countering the mechanisms by which tumors evade the immune system. Key effector cells, such as CD8^+^ effector T cells, are components of the immune system that identify and eliminate cancer cells by recognizing tumor-specific antigens (neoantigens). The activation of T cells is regulated by immune checkpoints [20]. In recent years, ICT has become a new option for patients with platinum-refractory or platinum-ineligible disease [6]. ICT targets CTLA4 and PD-1/PD-L1, which are utilized by tumor cells to suppress anti-cancer immune responses [7]. In patients with platinum-refractory bladder cancer, ICT has shown superior efficacy compared to second-line chemotherapy [21]. Currently, some PD-1/PD-L1 inhibitors have been approved by the FDA and EMA for use in first-line (platinum-free chemotherapy) and second-line chemotherapy (after platinum chemotherapy failure). Approved drugs that use PD-L1 inhibitors include atezolizumab and avelumab, and PD-1 inhibitors include nivolumab and pembrolizumab. PD-1/PD-L1 inhibitors, such as pembrolizumab and avelumab, restore T cell activity by blocking immune-inhibitory signals [22].

### 2.2. Potential Value of Metabolic Regulation

The dual metabolic reprogramming that occurs in tumor cells and immune cells is a key aspect affecting the anti-tumor immune response in the TME [23]. Tumor cells exhibit a preference for rapidly acquiring energy through the Warburg effect to support their high proliferation rate, while reducing oxidative stress [24]. Additionally, they produce excessive lactic acid, which further affects the phenotypic and functional characteristics of immune cells. Similarly, immune cells undergo different metabolic patterns during the transition from a steady state to activation, significantly affecting their proliferation, differentiation, and effector functions. Due to the mismatch between energy demands and nutrient availability, cancer cells and immune cells engage in metabolic competition, hindering the full potential of the immune response and accelerating tumor growth. Key enzymes involved in glycolysis play a dual role, supporting the efficient metabolism of tumor cells, while also regulating tumor initiation and development through non-metabolic mechanisms such as anti-apoptosis, immune escape mediation, and participation in signaling pathways [25]. In addition to causing cell acylation, lactate in the TME acts as an energy substrate shuttling between different cells, and researchers are gradually recognizing that lactate, as a signaling molecule, mediates crosstalk between metabolic pathways, immune responses, and intercellular communication within the TME. This creates a vicious cycle of metabolic reprogramming for effective anti-tumor immunity, with immune suppression promoting tumor growth, and tumor progression in turn exacerbating metabolic limitations [26]. Therefore, targeting metabolic pathways involved in glycolysis is a promising therapeutic strategy to reprogram the TME and restore immune function.

Lipids play an important role in cellular processes, including signal transduction, energy storage, and immune system function, making them an indispensable component of cell membranes. Lipid metabolism dysregulation is considered a significant metabolic aberration in cancer, which may promote tumor initiation, modulate treatment responses, facilitate recurrence, and promote ion and metastatic progression. The proliferation of tumor cells requires a large number of fatty acids to meet the requirements of cell membrane biosynthesis [27]. In general, tumor cells can adapt to lipid metabolism through various mechanisms to meet their high demand for lipids and ensure an adequate supply of the lipids required for their rapid proliferation. Therefore, interfering with these regulatory mechanisms may provide new strategies and potential therapeutic targets for cancer treatment. In the TME, the intense competition for nutrients between immune cells and tumor cells also leads to the reprogramming of lipid metabolism. Specifically, M2 macrophages and Tregs exhibit highly chemoactive lipid metabolism, and different immune cells exhibit different metabolic profiles in their resting and activated states [28].

Furthermore, the impact of lipid metabolism on variant morphological immune cells also varies. Regulating the lipid metabolism of these immune cells is crucial for maintaining their function and activity, and is a key approach to enhancing tumor immune efficacy.

## 3. Metabolic Reprogramming of Glucose and Immune Therapy Resistance in BCa

### 3.1. Characteristics of the Warburg Effect in Bca

**Figure 2:**
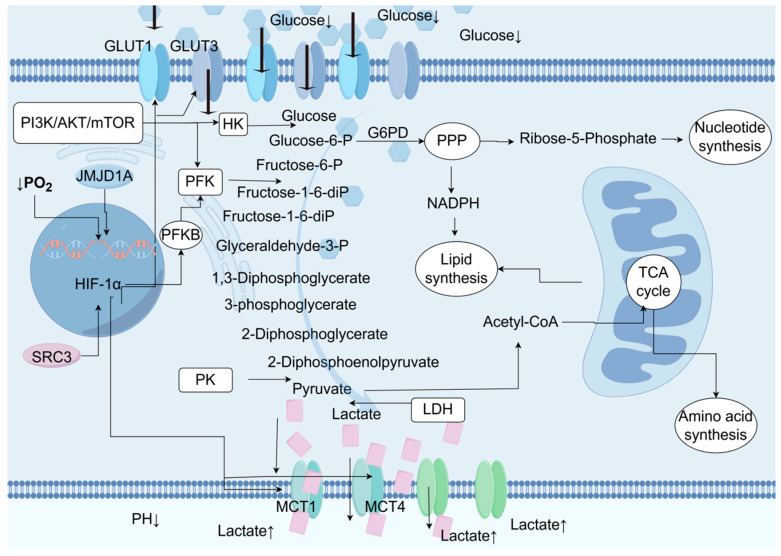
During the process of glucose metabolism reprogramming in urothelial carcinoma (UC), the oxygen-dependent hypoxia-inducible factor 1α (HIF-1α) and the oxygen-independent PI3K/AKT/mTOR signaling pathway drive the metabolic reprogramming in UC by regulating the activity of rate-limiting enzymes or transporters. Glucose is absorbed by cells through glucose transporters (GLUT) 1 and 3. The glycolysis process begins with hexokinase (HK) phosphorylating glucose to form glucose-6-phosphate, thereby preventing glucose from diffusing out of the cell. Glucose-6-phosphate can be dehydrogenated by glucose-6-phosphate dehydrogenase (G6PD) to enter the pentose phosphate pathway (PPP), producing pentoses such as ribose-5-phosphate for nucleotide synthesis, as well as the NADPH required for reduction processes like lipid biosynthesis. If glucose-6-phosphate is not oxidized by G6PD, it will be isomerized to fructose-6-phosphate by phosphoglucose isomerase (PGI). Subsequently, phosphofructokinase (PFK) catalyzes the phosphorylation of fructose-6-phosphate to form fructose-1,6-bisphosphate, irreversibly directing glucose-derived metabolites towards the glycolysis pathway, towards phosphoenolpyruvate. At the end of the glycolysis pathway, pyruvate kinase (PK) catalyzes the dephosphorylation of phosphoenolpyruvate (PEP) to produce pyruvate. Pyruvate is then metabolized into lactate by lactate dehydrogenase (LDH). Lactate is transported out of the cell by monocarboxylate transporters (MCT) 1 and 4, resulting in an increase in extracellular lactate concentration and acidification of the tumor microenvironment. Additionally, pyruvate can also be metabolized into acetyl-coenzyme A (acetyl-CoA), which participates in the mitochondrial tricarboxylic acid (TCA) cycle, producing ATP and intermediate metabolites that are essential for lipid and amino acid biosynthesis.

BCa cells exhibit significant aerobic glycolysis (the Warburg effect; Figure 2), preferentially utilizing glycolysis for energy production even under oxygen-rich conditions, sacrificing energy efficiency to obtain biosynthetic precursors (such as nucleotides and lipids). Enhanced glucose uptake in UC can be observed through PET/CT, and the glucose uptake and lactate production in UC cell lines are significantly higher than in normal urothelial cells [29]. The glucose content in UC tumor samples is significantly lower than in normal urothelial cells. Increased late intermediates of the TCA cycle suggest the replenishment of metabolic intermediates required for biosynthesis through anaplerosis. The pentose phosphate pathway is significantly upregulated in UC, generating NADPH (antioxidant) and ribose (nucleotide synthesis). TCA cycle intermediates are drawn for biosynthesis, and late TCA intermediates (such as malate and succinate) are increased in UC [29]. Glucose utilization in UC is regulated by both hypoxia-dependent and hypoxia-independent mechanisms, involving various glucose transporters and glycolytic enzymes. The hypoxia-dependent mechanism is mediated by the transcription factor hypoxia inducible factor-1α (HIF-1α), inducing the expression of glycolysis-related genes (such as GLUT1, LDHA, and MCT4), and inhibiting oxidative phosphorylation (through PDK1 inhibition of pyruvate dehydrogenase). Steroid receptor coactivator 3 (SRC-3) and histone demethylase JMJD1A are coactivators of HIF-1α, enhancing its transcriptional activity [30,31]. The hypoxia-independent mechanism is mainly mediated by the activation of the PI3K/AKT/mTOR pathway, which includes activating factors (PI3K, AKT, and mTOR) and inhibitory factors (PTEN). Increased glycolytic flux is associated with the upregulation of glucose transporter expression. Glucose transporter 1 (GLUT1) is the major overexpressed transporter in cancer. Glucose transporter 3 (GLUT3) expression is also detected in UC cells [32]. UC upregulates glycolytic flux through HIF-1α and neutralizes ROS through regulatory factors such as GLUT1/3, PFK, and MCT4. The PI3K/AKT/mTOR signal upregulates glycolytic enzymes, such as HK, to promote glycolysis.

### 3.2. The Immunosuppressive Mechanism Mediated by Glucose Metabolism in BCa

**Figure 3:**
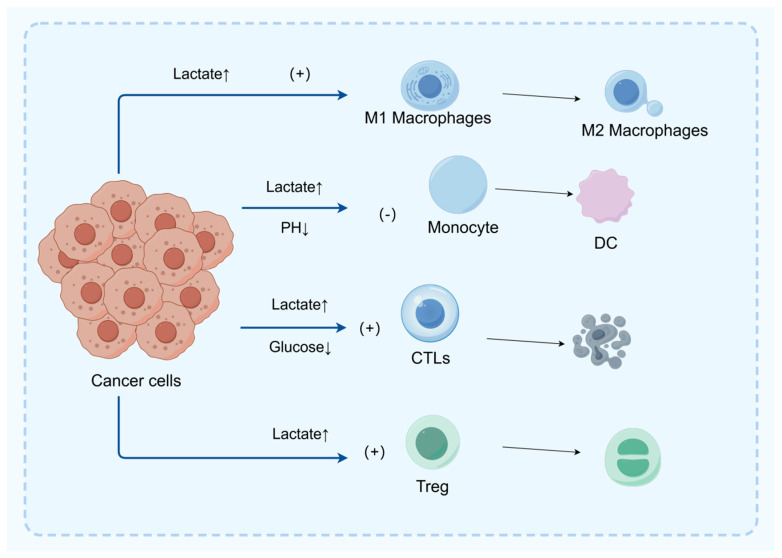
In the tumor microenvironment (TME), excessive lactic acid promotes the polarization of M1 macrophages to M2 macrophages, thereby suppressing the immune response and facilitating immune escape of tumor cells. Furthermore, lactic acid inhibits the migration and differentiation of monocytes into dendritic cells (DC), thus hindering the antigen presentation process and subsequent T cell activation. Lactic acid can also directly inhibit the function of CD8^+^ T cells and achieve immune suppression by promoting the proliferation of regulatory T cells (Treg).

As shown in Figure 2, the TME is a key factor affecting tumor behavior and treatment response. The increased metabolic activity of tumor cells leads to a decrease in the content of glucose and other nutrients in the TME, and low glucose utilization triggers metabolic competition between effector T cells and tumor cells. This phenomenon alters the effector function of tumor-infiltrating lymphocytes and the immune response to the tumor [33] (Figure 3).

BCa cells produce excessive lactic acid (TME concentration can reach 20–40 mM) through the Warburg effect, which enters CD8^+^ T cells via monocarboxylate transporters (MCT1/4), leading to intracellular acidification (pH drops to 6.5–6.8), inhibiting the mTORC1 signaling pathway and glycolytic enzyme activity, and weakening T cell proliferation and effector function. In a melanoma model [34], LDHA inhibitors reduce TME lactic acid concentration, increase CD8^+^ T cell infiltration by 2 times, restore IFN-γ secretion by 70%, and significantly prolong survival when combined with anti-PD-1 therapy. Single-cell sequencing reveals [35] that in BCA patients, CD8^+^ T cells in high lactic acid areas have downregulated expression of glycolytic genes (such as GLUT1 and HK2) and upregulated expression of oxidative phosphorylation genes (such as COX5B), suggesting impaired metabolic adaptability.

#### 3.2.1. Promoting the Expansion of Regulatory T Cells (Treg)

Lactic acid activates the GPR81 receptor, inducing the expression of Foxp3 and CTLA-4 in Treg cells, while enhancing the transcription of immunosuppressive genes (IL-10, TGF-β) through epigenetic modifications (such as H3K27ac acetylation). In BCa patients [36], TME lactic acid concentration is positively correlated with the proportion of Treg; knocking out the GPR81 gene can reduce Treg infiltration (by 40%) and enhance the efficacy of anti-PD-L1 therapy (tumor volume reduction by 55%). Lactic acid stabilizes the epigenetic characteristics of Treg by inhibiting TET2-mediated DNA demethylation [37]. In preclinical models, lactic acid scavengers (such as lactate oxidase) can reverse Treg-mediated immunosuppression.

#### 3.2.2. Driving M2 Macrophage Polarization

Tumor-associated macrophages (TAMs) are generally considered to promote tumor progression and metastasis. However, TAMs exhibit a dual function, including pro-inflammatory M1 macrophages with anti-tumor activity and M2 macrophages that promote tumor growth and immune escape. Studies have shown that lactate produced by tumor cells can upregulate the expression of HIF-1α in TAMs, inducing polarization from the M1 to the M2 phenotype [38,39].

Lactic acid activates the HIF-1α/STAT3 pathway within macrophages, upregulating arginase-1 (Arg1) and IL-10 expression while suppressing iNOS and TNF-α production and promoting M2 polarization. Single-cell RNA sequencing shows [40] that in bladder cancer M2 macrophages, lactic acid metabolism genes (MCT1, LDHA) are highly expressed and negatively correlated with overall patient survival. Lactic acid activates the AMPK-ULK1 axis to induce macrophage autophagy, promoting M2 phenotype transformation; AMPK inhibitors (such as Dorsomorphin) can reverse this effect, increasing the M1/M2 ratio from 1:4 to 1:1 [37].

#### 3.2.3. Inhibiting Dendritic Cell (DC) Function

Acidic TME (pH ≤ 6.8) inhibits the antigen-presenting ability of DCs, first by downregulating MHC-II expression, and lactic acid inhibits NF-κB signaling, reducing CIITA (MHC-II transcription activator) expression. Second, there is a migration disorder, as the low-pH environment disrupts the CCR7-CCL19 chemokine axis, hindering DC migration to lymph nodes. In a BCa model [33], neutralizing TME acidity (oral sodium bicarbonate) restores MHC-II expression in DCs by 60%, and lymph node T cell activation rates increase by two times. Lactic acid induces mitochondrial oxidative stress in DCs [41], inhibiting ATP synthesis, and in clinical samples, DC mitochondrial membrane potential is positively correlated with patient immunotherapy response.

#### 3.2.4. Metabolic Competition Leading to T Cell Exhaustion

Tumor cells excessively uptake glucose through GLUT1 (uptake rate is 20 times higher than normal cells), causing TME glucose concentration to drop to 0.1–0.5 mM, forcing CD8^+^ T cells to switch to fatty acid oxidation for energy, but their mitochondrial metabolic capacity is insufficient, ultimately leading to functional exhaustion [42]. Lv et al. reported that circFAM13B could repress immune evasion and enhance immunotherapy sensitivity by inhibiting glycolysis and promoting CD8^+^ T cell function in BCa [43].

## 4. Clinical Translation Strategies Targeting Glucose Metabolism

Currently, clinical trials are dedicated to combining ICI with drug therapies targeting tumor metabolic pathways.

Studies indicate that in certain patient populations, resistance to ICI is mainly influenced by the concentration of lactates in the tumor microenvironment. The presence of lactate aids in the survival and function of immune suppressive cells within the microenvironment, while also promoting an increase in the expression of PD-L1 on the surface of tumor cells. Recent research has revealed that the upregulation of PD-L1 expression in nasopharyngeal carcinoma cells is closely related to the process of active glycolysis. A drug originally used for the treatment of hepatitis, cirrhosis, and liver protection can interfere with the glycolytic pathway mediated by HIF-1α/LDHA, prompting cellular metabolism to shift towards mitochondrial oxidative phosphorylation, thereby reducing the expression level of PD-L1 [44].

LDHA increases the infiltration of activated CD8^+^ T cells in tumors and also enhances the therapeutic effect of pembrolizumab [45]. Furthermore, studies have shown that the combination of LDHA inhibitors with anti-PD-1 therapy can significantly reduce the tumor volume in a mouse orthotopic BCa model compared to the use of anti-PD-1 therapy alone [46].

Metformin, as a drug clinically used to treat type 2 diabetes, involves a mechanism of action that interferes with the HIF-1 signaling pathway, thereby affecting the glycolysis process of various cancers [47,48]. Studies indicate that metformin can significantly improve the immune microenvironment of patients with type 2 diabetes and colorectal cancer [49]. Furthermore, the combined application of metformin and immune checkpoint inhibitors has shown enhanced efficacy in the treatment of melanoma [50]. However, other studies suggest that metformin may inhibit the sensitivity of tumor cells to paclitaxel by inducing lactic acidosis [51]. In light of this, whether metformin can improve the tumor microenvironment by targeting metabolic pathways and thereby enhance the effects of immunotherapy still needs to be verified through systematic studies.

It is noteworthy that active components in natural products can directly act on the glycolytic pathway and PD-L1, or activate anti-tumor immune responses through the glycolytic pathway. For instance, ginsenoside Rh4 in ginseng regulates the AKT/mTOR signaling pathway, inhibits glycolysis, and reduces PD-L1 expression, thereby enhancing anti-tumor immune responses [52]. Studies have found that specific natural polyphenol compounds present in grapes and berries can increase the sensitivity of ovarian cancer to immunotherapy by inhibiting the glycolytic process [53]. Various bioactive components in traditional Chinese medicine [54], including quinones, phenols, flavonoids, and terpenoids, exhibit the potential to inhibit tumor cell proliferation, metastasis, and invasion by interfering with the glycolytic process. These natural compounds with different mechanisms of action provide important perspectives for research in the field of tumor metabolism–immunotherapy. For strategies targeting glycolytic metabolism, we believe two aspects need to be considered: one is to enhance the effects of anti-PD-L1/PD-1 therapy by altering the glucose metabolism of non-tumor cells such as immune cells and tumor vascular endothelial cells; the other is to directly act on the glycolytic process of tumor cells to improve the efficacy of PD-L1/PD-1 therapy.

## 5. Lipid Metabolism Reprogramming and Immunotherapy Resistance in Bca

**Figure 4:**
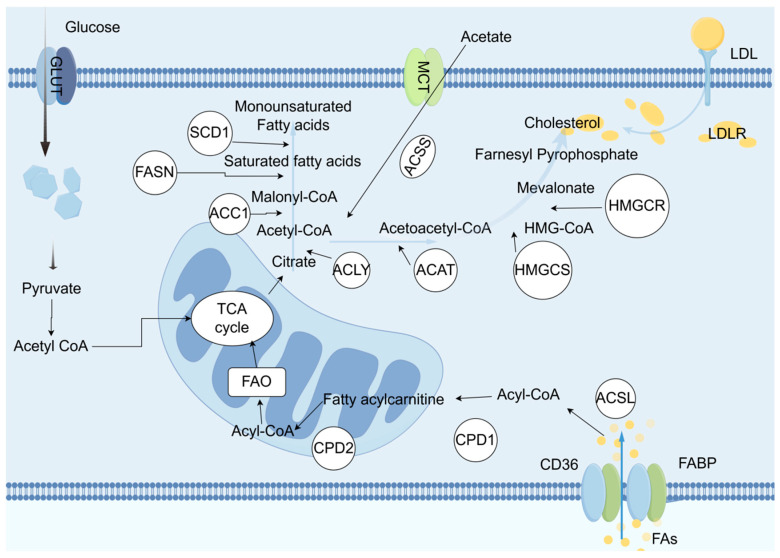
In malignant cells, the biosynthesis of fatty acids and cholesterol both use acetyl-CoA as the starting substrate. Acetyl-CoA is carboxylated by the enzyme ACC1 to form malonyl-CoA, which then undergoes a multi-step condensation reaction catalyzed by fatty acid synthase (FASN), resulting in the production of a 16-carbon saturated fatty acid. Subsequently, under the action of stearoyl-CoA desaturase (SCD1), the saturated fatty acid undergoes desaturation to be converted into a monounsaturated fatty acid. The biosynthesis of cholesterol relies on the mevalonate pathway, where acetyl-CoA undergoes a series of transformation steps to form 3-hydroxy-3-methylglutaryl-CoA (HMG-CoA), which is then reduced to mevalonate by HMG-CoA reductase (HMGCR), ultimately synthesizing cholesterol through a series of complex biochemical reactions. Furthermore, during the proliferation and development of tumor cells, the expression of fatty acid transport proteins such as CD36 and fatty acid-binding proteins (FABP) is upregulated, enhancing the activity of FASN, thereby accelerating the uptake of fatty acids and cholesterol. In summary, tumor cells regulate lipid metabolism through various mechanisms to meet their high demand for lipids and ensure an adequate supply of lipids required for their rapid proliferation. Therefore, disrupting these regulatory mechanisms may provide new strategies and potential therapeutic targets for cancer treatment.

With the development of metabolomics detection technology, various lipid metabolites with abnormal content have been found in bladder cancer tissues, blood, or urine samples. Compared with normal bladder tissues, bladder cancer tissues have higher levels of phospholipids and fatty acids, while triglyceride levels are lower. Additionally, the content of diacylglycerol increases with the progression of tumor staging [55]. Another study showed that compared with normal urothelium, multiple lipids are significantly upregulated in urothelial carcinoma, including medium-chain, long-chain, polyunsaturated fatty acids, and branched-chain fatty acids, glycerophospholipids, and sphingolipids. A study that determined the fatty acid composition in tissues also confirmed that bladder cancer tissues have higher contents of stearic acid and oleic acid [56]. In a metabolomics study using plasma samples, the results showed that compared with plasma from healthy volunteers, there are differences in the content of specific metabolites related to the pentose phosphate pathway, nucleotides, and fatty acid synthesis in the plasma of patients with high-grade bladder cancer [57]. A study [58] conducted metabolomics analysis on urine samples from bladder cancer patients, revealing nearly 1000 different metabolic feature molecules, and found that metabolites such as lactate, succinate, and palmitoyl sphingomyelin are different in the urine of bladder cancer and non-cancer patients. Furthermore, a tracer metabolomics method based on nuclear magnetic resonance revealed an increase in fatty acid synthesis in cisplatin-resistant bladder cancer cells (Figure 4).

The lipid metabolism reprogramming in BCa reshapes the TME and drives immune suppression by regulating fatty acid synthesis, oxidation, and cholesterol metabolism. Tumor cells and CD8^+^ T cells compete for fatty acid uptake [59]. Depleting fatty acids in the TME hinders the infiltration and function of CD8^+^ T cells [60].

### 5.1. Enhanced Fatty Acid Synthesis and Immune Evasion

In BCa cells, the expression of fatty acid synthase (FASN) and acetyl-CoA carboxylase is upregulated, promoting de novo synthesis of fatty acids (FA), generating immunosuppressive lipids (such as prostaglandin E2, PGE2), and suppressing CD8^+^ T cell function. Lipidomic analysis has found that the level of PGE2 in metastatic BCa tissues is higher than in normal tissues; inhibiting COX-2 (PGE2 synthase) can reverse T cell exhaustion, restoring IFN-γ secretion [61]. We found that circZNF609 inhibited BCa immunotherapy sensitivity via enhancing fatty acid uptake through the IGF2BP2/CD36 pathway [62].

### 5.2. Cholesterol Metabolism Imbalance and T Cell Function Inhibition

In various types of cancer, the significant accumulation of cholesteryl esters has attracted widespread attention. The fundamental cause of this phenomenon lies in the dysregulation of cholesterol homeostasis within cancer cells, primarily characterized by an increase in the biosynthesis or uptake of cholesterol, and a reduction in the efflux process of cholesterol [63]. In a study on prostate cancer [64], it was found that PTEN loss promotes the transcription of the SQLE gene by activating SREBP-2, thereby upregulating the expression of squalene epoxidase (SQLE). Furthermore, PTEN loss also enhances the stability of the SQLE protein by inhibiting the PI3K/Akt/GSK3β-mediated proteasome pathway, thus promoting the biosynthesis of cholesterol. The oncogene KRAS significantly affects the cholesterol metabolism of cancer cells by inducing SREBP-2 and subsequently promoting the transcription of the HMGCR gene. In triple-negative breast cancer [65], the EGFR-MAPK signaling pathway regulates the expression of the low-density lipoprotein receptor (LDLR), which acts as a key molecule in mediating the uptake of cholesterol. LDLR can specifically recognize and bind to low-density lipoprotein (LDL), forming a complex that is internalized into vesicles. Inside the vesicles, LDL is broken down, releasing cholesterol and other lipid components. Therefore, the upregulation of LDLR expression levels leads to an increase in cholesterol uptake, which in turn promotes the proliferation and migration of cancer cells, accelerating the cancer process. In bladder cancer tissues [66], the level of 25-hydroxycholesterol is elevated. As a product of cholesterol oxidation, it can promote the proliferation of human bladder cancer cells and epithelial–mesenchymal transition.

In the high-cholesterol environment infiltrated by tumors, the excessive accumulation of cholesterol in CD8^+^ T cells induces the unfolded protein response in the endoplasmic reticulum, resulting in endoplasmic reticulum stress. At the same time, this environment also induces T cell exhaustion by upregulating the expression of immune checkpoint molecules on the surface of CD8^+^ T cells, allowing tumor cells to evade immune effects [67].

### 5.3. Lipid Droplet Accumulation and Ferroptosis Resistance

Tumor cells store polyunsaturated fatty acids through lipid droplets to avoid ferroptosis caused by lipid peroxidation. Cell cycle arrest effectively inhibits ferroptosis. Mechanistically, cell cycle arrest induces diacylglycerol acyltransferase-dependent lipid droplet formation to sequester excess polyunsaturated fatty acids. These polyunsaturated fatty acids accumulate in triacylglycerols within arrested cells, thereby inhibiting ferroptosis [68].

## 6. Clinical Translation Strategies Targeting Lipid Metabolism

In some in vivo and in vitro studies related to FASN, it was found that FASN promoted resistance to gemcitabine in BCa. The use of its inhibitor (TVB-3166) can reverse this resistance effect in vitro or in vivo [69]. Some studies have pointed out that ACAT1 is highly expressed in BCa tissues, and is associated with a high pathological grade and poor clinical prognosis. The ACAT inhibitor avasimibe can inhibit the cell viability and proliferation of BCa and can also suppress the migration of Bca cells, while increasing ROS stress to induce cell cycle arrest in cancer cells [70]. The natural product cerulenin, an inhibitor of fatty acid synthase [71], can inhibit the proliferation and migration of bladder cancer cells [72]. FASN-targeted drugs, such as orlistat, can reverse the resistance of bladder cancer models to programmed death-1 (PD-1) therapy [73]. The use of simvastatin can increase the sensitivity of in vitro bladder cancer resistance models to doxorubicin. At the same time, an increasing amount of research evidence indicates that the response of cancer cells to treatment is controlled by their metabolic state, suggesting that tumor cell metabolism-related pathways can overcome their resistance by controlling the metabolic state [74].

## 7. Summary and Outlook

The metabolic reprogramming and immune therapy resistance mechanisms of BCa have gradually become clearer. There are mainly two aspects. One is the immunosuppression driven by glucose metabolism: the Warburg effect significantly weakens CD8^+^ T cell function through lactate accumulation, the acidic microenvironment, and Treg expansion. The other is the remodeling of the immune microenvironment by lipid metabolism: enhanced fatty acid synthesis leads to the accumulation of immunosuppressive lipids such as PGE2, while cholesterol metabolism imbalance directly damages T cell function. Inhibiting FASN or ACAT1 can enhance the efficacy of ICIs.

In recent years, there have been some studies on targeted drugs for glucose metabolism and lipid metabolism in bladder cancer (Table 1). Future research needs to deepen the study of mechanisms, as the interaction between metabolism and immunity has a spatiotemporal dynamic nature. Most existing studies are based on static metabolomic data and need to be combined with single-cell spatial transcriptomics and in vivo imaging techniques to analyze the real-time distribution of metabolic products in the TME and the dynamic association with immune cell function. Metabolic tracing techniques (such as dynamic tracking of ^13^C-glucose) can clarify the transfer path of lactate between T cell subsets and its impact on the formation of immune synapses. There is metabolic heterogeneity among individuals, and the metabolic heterogeneity of BCa may be driven by epigenetic regulation (such as m^6^A modification-regulating GLUT1 expression) or tumor stem cell characteristics. Key genes regulating metabolic plasticity need to be identified through organoid models and CRISPR screening. Existing metabolic inhibitors (such as FASN inhibitors) are often limited in clinical application due to toxicity to normal tissues. Developing tumor-specific delivery systems (such as nanoparticles targeting tumor cells) or prodrug design (such as LDHA inhibitors activated in a low-pH microenvironment) is an important research direction. For the optimization of the biomarker system, a single biomarker has limited predictive value, and a multimodal predictive model needs to be constructed (integrating ctDNA mutations, metabolic imaging omics features, and immune cell infiltration scores). For example, PET-CT combined with ^18^F-FDG (glucose metabolism) and ^11^C-acetate (lipid metabolism) imaging can non-invasively assess tumor metabolic activity.

## 8. Conclusions

Future BCa treatment needs to break through the traditional “single-target” thinking and instead focus on multi-node intervention in the metabolism–immunity interaction network. For example, studies could consider a triple therapy design, simultaneously targeting glucose metabolism (LDHA inhibitor), lipid metabolism (FASN inhibitor), and immune checkpoints (anti-PD-1), and remodeling the “hot tumor” microenvironment through metabolic reprogramming. Artificial intelligence could also be used to assist decision-making, using machine learning to integrate multi-omics data (metabolomics, immunomics, genomics) and predict the best combination scheme and dynamically optimize the dose (such as adaptive treatment guided by reinforcement learning algorithms).

## Figures and Tables

**Table 1:** 2023–2025 targeted drug research summary table for bladder cancer glucose metabolism and lipid metabolism. Adapted from [75,76,77,78].

Drug/Therapy	Target	Mechanism of Action	Research Stage	Main Research Results
Simvastatin	Mevalonate pathway	Inhibits HMG-CoA reductase, reduces cholesterol synthesis, and targets bladder cancer cells with high NFYC-37 expression.	Preclinical (animal model)	Significantly inhibits tumor growth in mouse models, associated with NFYC-37 splicing variants.
Melatonin	PPARγ/ENO1	Inhibits the PPARγ/ENO1 pathway, reduces glycolysis, and enhances the chemosensitivity of gemcitabine.	Preclinical (cells/animals)	Inhibits glucose uptake and lactate production in bladder cancer cells, induces ROS accumulation, and has a significant synergistic effect with chemotherapy.
2-Deoxyglucose (2DG)	HK2	Inhibits hexokinase 2, blocks glycolysis, and enhances the chemosensitivity of cisplatin.	Preclinical (cells/animals)	Reduces the survival rate, migration, and invasion ability of bladder cancer cells. The combined treatment with cisplatin significantly inhibits tumor growth.
Metformin	SREBP-1c/FASN	Inhibits the SREBP-1c/FASN axis through downregulation, reduces fatty acid synthesis, and enhances the anti-tumor effect.	Preclinical (cells/animals)	Downregulates the expression of FASN, inhibits the proliferation of bladder cancer cells, and has a significant effect when combined with chemotherapy.

## Data Availability

Data available in a publicly accessible repository.
